# Some Advantages of a Declining Birth-Rate

**Published:** 1909-04-24

**Authors:** 


### Public Health and Hygiene.

### SOME ADVANTAGES OF A DECLINING BIRTH-RATE.

It is the fashion—a fashion doubtless based upon sincerity of purpose and conviction—to lament the decline in national fertility disclosed by the statistical returns of registered births. Each year the annual report of the Registrar-General records a further drop in the birth-rate and a practically unbroken process of continued decline to which it is subject. Now a statistical phenomenon is a mere abstraction of the barest content; it almost never in itself can be an object either of approval or opprobrium.

It is not self-evident that the fact of a declining birth-rate is an occasion for regret. Take the population of the world, or any natural division or artificially selected portion of it, as a closed social system, and it is evident that its state of well-being may demand, according to circumstances, either an increase or a diminution of its numerical proportions; or it may be that a balance has been reached in which, having regard to all the conditions, no benefit could result from either change. A stationary, advancing, or receding birth-rate would be variously judged accordingly as one or other of these stages in such a social system had been attained.

These speculative considerations, although the fact is not always appreciated, underlie any judgment as to the value of the increase or decline of the birth-rate. In considering the birth-rate, therefore, we are carried at once beyond the arithmetical expression to the actual facts which constitute the matter of real concern.

In dealing with the English birth-rate, we must inquire, first of all, whether it is necessary or desirable for the national well-being that the rate of increase to which we have become accustomed should continue. And this is a difficult and complex question to which an answer will not readily be forthcoming. Eugenic, political, economical, and many other considerations into which we cannot enter here, have to be taken into account before either an affirmative or negative reply is given.

Leaving this important aspect, we may with advantage inquire into the causes which are operative in bringing about a result as to the main value of which we suspend judgment.

The Registrar-General has for several years in his annual report given a table showing the movement of the birth-rate in relation to the number of women living at procreative age periods in England and Wales. These results as summarised in the annual report of 1907 are for 1876-1905:—

| | Birth-Rate per 1,000 females age 15-45 years. | | Birth-rate per 1,000 females age 15-45 years. |
|---|---|---|---|
| 1876-1880 ... | 153.3 | 1891-1895 ... | 126.8 |
| 1881-1885 ... | 144.3 | 1896-1900 ... | 118.8 |
| 1886-1890 ... | 133.4 | 1901-1905 ... | 112.5 |

From this table it is clearly seen that the decline in the birth-rate is due to a diminished fertility of women capable of child-bearing. When we turn to the Registrar-General's statistics of illegitimate births, we find that in 30 years the birth-rate of illegitimates has declined from 14.4 per 1,000 unmarried or widowed females at procreative age periods in 1878 to 7.8 per 1,000 in 1907. So far as the general decline in the birth-rate is to be ascribed to this contributory cause it must be contemplated with satisfaction. Again, when we consider ages at marriage we find that there has been a marked increase in the age at which women enter into matrimony, and that as a result the number of years spent by women of child-bearing age in wedlock has been proportionately diminished. So far as this directly affects the birth-rate there will be few who will regret the loss resulting from a cause which has been accepted as a desirable change.

We learn from the Registrar-General's report, however, that the operation of these combined causes accounts for only 21 per cent. of the decline in the birth-rate. "There are sufficient grounds for stating that during the past 30 years approximately 14 per cent. of the decline in the birth-rate (based on the proportion of births to the female population aged 15-45 years) is due to the decrease in the proportion of married women in the female population of conceptive ages, and that over 7 per cent. is due to decrease of illegitimacy. With regard to the remaining 79 per cent. of the decrease, although some of the reduced fertility may be ascribed to changes in the age constitution of married women, there can be little doubt that much of it is due to deliberate restriction of child-bearing. The fact is also significant that at the last Census period, 1900-02, the fertility of English wives was lower than that recorded in any European country except France."

It is this "deliberate restriction of child-bearing" among English wives which has called forth the fulminations to which we have referred above. Although upon the whole question we are in no position to dogmatise, there is an aspect of it to which as hygienists we feel constrained to call attention. We consider a high infantile mortality as hygienically and economically unsound; and infantile mortality is only an extreme expression of inefficient breeding and nurture. The unaided mother of a family, even when she is fortunate enough to escape being a contributory wage-earner, can rarely do justice to the numerous progeny which she is naturally capable of bearing. If restricted child-bearing is compensated in improved mothering of a fewer offspring, and if smaller families mean a higher individual fostering and culture, a greater personal attainment and efficiency, the numerical decrease in the growth of population, most people will agree, will not be a high price for the purchase of so desirable a result. And we suspect that if it could be and were investigated, it would be found that this is the true meaning of the statistical phenomenon which is deplored. What we wish to insist upon is that it may or may not be deplorable, that conceivably it may be ground for gratification, and that in any case judgment must be suspended until the meaning of the phenomenon is made clear.

